# Successful Management of Jejunal Perforation in Burkitt's Lymphoma: A Case Report

**DOI:** 10.1155/2012/230538

**Published:** 2012-09-17

**Authors:** David A. Finch, Malcolm S. Wilson, Sarah T. O'Dwyer

**Affiliations:** Department of Surgical Oncology, The Christie Hospital, Wilmslow Road, Manchester M20 4BX, UK

## Abstract

Burkitt's lymphoma (BL) is rare, rapidly growing, and highly aggressive. Urgent commencement of chemotherapy is vital to prevent complications and promote a favourable prognosis. Any factor causing a delay in the initiation of chemotherapy will affect prognosis significantly. Intestinal perforation is a well-known complication with devastating consequences. It inevitably leads to a delay in the initiation of chemotherapy. There are few reports in the literature that discuss this complication. Furthermore, there are no reports of patients that have survived intestinal perforation occurring prior to the commencement of chemotherapy. We present a case of a 55-year-old male who survived perforation of advanced sporadic BL of the jejunum occurring prior to the commencement of chemotherapy. Critical aspects of the patients care are discussed.

## 1. Introduction

Burkitt's lymphoma (BL) is a rare, high-grade B-cell non-Hodgkin's lymphoma, it is rapidly growing and highly aggressive. With the potential for a tumour to double in size every 18 hours [1], it is one of the fastest growing human malignancies. Urgent commencement of chemotherapy is vital. The presence of sepsis or any other condition that results in the delay of chemotherapy treatment will worsen the prognosis significantly. We present a case of advanced sporadic BL of the jejunum complicated by sepsis secondary to jejunal perforation.

## 2. Case Report

A fifty-five-year-old man was admitted for chemotherapy following a diagnosis of Burkitt's lymphoma on a three-month background of increasing abdominal pain, severe night sweats and a four-kilogram weight loss. He had no significant past medical history.

**Figure 1:**
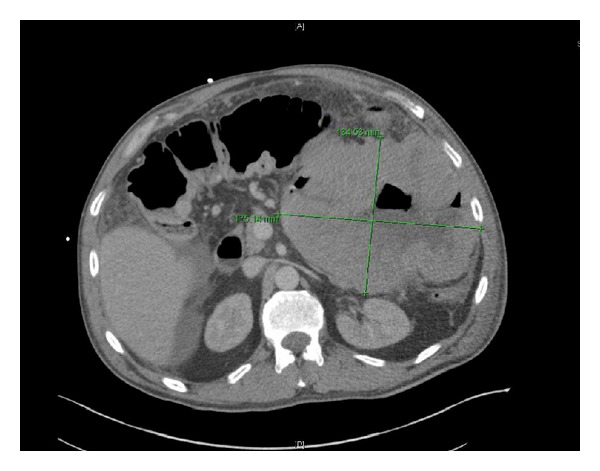


Prior to commencing chemotherapy, the patient developed peritonitis. An abdomino-pelvic CT scan demonstrated extensive small bowel lymphoma with a mass measuring 21 × 17 × 13 cm and a pneumoperitoneum indicating bowel perforation (Figure 1).

After a twelve-hour period of critical care input with invasive monitoring, vasopressin, and respiratory support, a laparotomy was undertaken. Findings revealed gross peritonitis secondary to perforated mid small bowel due to a large unresectable tumour mass.

The abdomen was washed out and drained. In order to defunction the midgut, a venting gastrostomy was inserted and a loop jejunostomy was formed distal to the diseased segment approximately one and a half metres from the ileocaecal valve.

Accepting the potential risks of sepsis, further perforation and tumour lysis syndrome [2], cyclophosphamide, doxorubicin, vincristine and prednisolone (CHOP) chemotherapy were given at fifty percent of regular dosing day one postoperatively under antibiotic cover with tazocin and vancomycin. Antibiotics were discontinued once inflammatory markers stabilised and neutropenia resolved.

A coordinated multidisciplinary approach over the ensuing eight weeks from surgeons, medical oncologists, microbiologists, and support teams ensured the patient remained fit enough to undertake full-dose cyclophosphamide, vincristine, doxorubicin, methotrexate, rituximab, ifosfamide, cytarabine, etoposide, and intrathecal methotrexate (CODOX-M/RIVAC) chemotherapy.

Initial nutritional support was through the intravenous route. As soon as was possible, nutrition was given by enteroclysis via the loop jejunostomy in order to reduce the risk of sepsis and to establish bowel function [3]. Input from the Dietetic and the Palliative Care Teams was vital in order to optimise his nutrition and to provide him with effective analgesia and antiemetics. Weekly psychooncological review offered further support.

Two episodes of neutropenic sepsis were again managed with intravenous broad-spectrum antibiotics along withgranulocyte colony-stimulating factor (GCSF) and blood/platelet transfusions. Cultures proved negative on all occasions.

**Figure 2:**
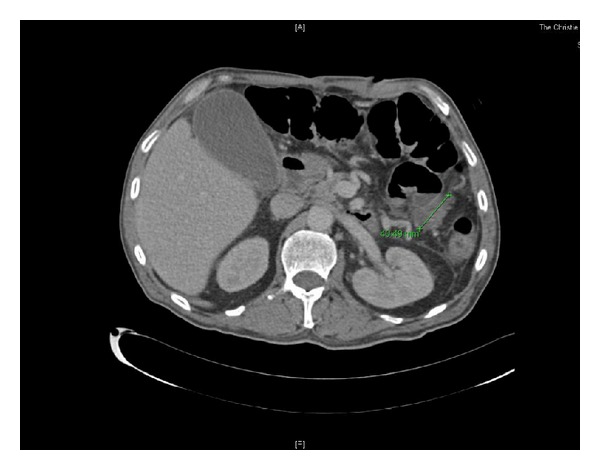


A suspicion of a residual abdominal collection led to a CT abdomen and pelvis being performed following the third cycle of chemotherapy. Whilst no evidence of collection was seen, extensive pneumatosis intestinalis involving the right and transverse colon as a result of neutropenia indicated neutropenic colitis. As there were no signs of peritonism, the patient was managed conservatively with tazocin and GCSF [4]. Additionally, this scan showed dramatic tumour response with the tumour now only measuring 4 × 2.4 cm (Figure 2).

PET CT following the fourth and final cycle of the CODOX-M/RIVAC regimen indicated a complete tumour response. Six weeks after the final cycle of chemotherapy, the patient underwent surgery to restore gastrointestinal continuity. A section of proximal jejunum including the perforated segment and biopsies taken at this time displayed no evidence of residual neoplasm.

The patient remained in hospital for the duration of his care through to the restoration of gastrointestinal continuity. At six months post completion of treatment, the patient is recovering well with no clinical or radiological signs of recurrence.

## 3. Discussion

Burkitt's lymphoma is predominantly seen in children but rarely may also present in adults. The majority of patients are male with a 3 or 4 : 1 male : female ratio [5, 6]. Three distinct clinical forms are recognised: endemic, sporadic, and immunodeficiency related.

The endemic variant mainly occurs in equatorial Africa. It is the most common malignancy of children in this area and is associated with chronic malaria and Epstein-Barr virus (EBV). It usually presents as a jaw or facial bone tumour. Sporadic BL is rare, with 2-3 cases per million per year [7], it is typically seen in the United States and Western Europe. Also associated with EBV but to a lesser degree, it usually has an abdominal presentation. Virtually all adult BL is of the sporadic form. Immunodeficiency-related BL is usually seen associated with HIV infection or the use of immunosuppressive drugs. B symptoms may be present with all forms and include fever, weight loss, and night sweats.

Biopsy is needed for diagnosis. Histology reveals monomorphic, medium-sized cells with basophilic cytoplasm, and a high proliferation fraction with the Ki-67 fraction approaching 100 percent.

Burkitt's lymphoma, whilst potentially very aggressive, is usually highly sensitive to chemotherapy [8]. The standard of care has yet to be defined, however, treatment of choice usually involves a short, intense combination of chemotherapy drugs. Complete remission rates for both early and advanced disease in adults of between 75% and 90% have been achieved [9, 10].

Complications of these treatments are, however, a serious concern. Profound neutropenia and related haematological toxicities leave patients susceptible to neutropenic sepsis. Tumour lysis syndrome can lead to the development of acute renal failure. These complications must be dealt with urgently and effectively to minimise disruption to the chemotherapeutic regimen.

Owing to the availability of such highly effective chemotherapeutic agents, the role for surgery is now typically limited to the management of complications of BL. In this case, it was necessary to deal with the peritonitis primarily followed by the rapid introduction of systemic treatment. This approach was in contrast to a recent case report of jejunal perforation in BL whereby chemotherapy was delayed postoperatively, the patient failed to survive [11]. Retrospectively it was thought that the outcome for the patient in question may have been different had chemotherapy been commenced in the early postoperative period.

Acting as a bridge to more intensive chemotherapy, CHOP chemotherapy permitted combination chemotherapy to be administered immediately in the day-one postoperative period. At fifty percent regular dosing and under the cover of broad-spectrum antibiotics, this helped balance the risks of potentiating abdominal sepsis versus the benefits of halting tumour growth.

A further complication of chemotherapy to consider in BL is chemotherapy-induced intestinal perforation, there is only one reported case of survival following this complication [12]. The two-stage surgical approach used in this case reduced the risks from further chemotherapy-induced perforation by defunctioning the diseased segment of bowel. Additionally, should there have been an inadequate response to chemotherapy, then the initial procedure would also have served as a palliative option. 

No attempt to resect disease was made. Resection of diseased bowel is not imperative and indeed before advances in chemotherapy when abdominal BL was treated with surgical cytoreduction and then followed up with chemotherapy postoperatively, patients were susceptible to further complications including anastomotic leak [13, 14].

In conclusion, BL is a rapidly progressive malignancy that may present with surgical complications. To our knowledge, there are no reports in the literature of patients surviving intestinal perforation in BL occurring prior to the commencement of chemotherapy. The rarity of such situations means guidelines for management of this complication and initiation of chemotherapy postoperatively are lacking.

When complicated by intestinal perforation, initial surgery should aim to create an environment which minimises further risks from chemotherapy-induced complications. Resection of disease is not essential. Postoperatively, efforts should be made for early chemotherapy in an attempt to reverse tumour growth and promote a favourable prognosis. Removal of potential sources of sepsis at the earliest opportunity, a high vigilance for complications of chemotherapy, and a low threshold for intervention all aid this approach. Multidisciplinary team involvement throughout is vital.

## References

[1] Phillips JA (2006). Is Burkitt’s lymphoma sexy enough?. *Lancet*.

[2] Davidson MB, Thakkar S, Hix JK, Bhandarkar ND, Wong A, Schreiber MJ (2004). Pathophysiology, clinical consequences, and treatment of tumor lysis syndrome. *American Journal of Medicine*.

[3] Zaloga GP (2006). Parenteral nutrition in adult inpatients with functioning gastrointestinal tracts: assessment of outcomes. *Lancet*.

[4] Urbach DR, Rostein OD (1999). Typhilitis. *Canadian Journal of Surgery*.

[5] Boerma EG, Van Imhoff GW, Appel IM, Veeger NJGM, Kluin PM, Kluin-Nelemans JC (2004). Gender and age-related differences in Burkitt lymphoma—epidemiological and clinical data from the Netherlands. *European Journal of Cancer*.

[6] Smith A, Howell D, Patmore R (2011). Incidence of haematological malignancy by subtype: a report from the Haematological Malignancy Research Network. *British Journal of Cancer*.

[7] Parente F, Anderloni A, Greco S, Zerbi P, Porro GB (2004). Image of the month. *Gastroenterology*.

[8] Ferry JA (2006). Burkitt’s lymphoma: clinicopathologic features and differential diagnosis. *Oncologist*.

[9] Todeschini G, Tecchio C, Degani D (1997). Eighty-one percent event-free survival in advanced Burkitt’s lymphoma/leukemia: no differences in outcome between pediatric and adult patients treated with the same intensive pediatric protocol. *Annals of Oncology*.

[10] Mead GM, Sydes MR, Walewski J, Grigg A, Hatton CS, Pescosta N (2002). UKLG LY06 collaborators. An international evaluation of CODOXM and CODOX-M alternating with IVAC in adult Burkitt’s lymphoma: results of United Kingdom Lymphoma Group LY06 study. *Annals of Oncology*.

[11] Rao J, Lee KC, Chan L, Chuah KL, Chiu MT (2008). Advanced Burkitt’s lymphoma presenting with jejunal perforation. *Annals of the Academy of Medicine Singapore*.

[12] Goldberg SR, Godder K, Lanning DA (2007). Successful treatment of a bowel perforation after chemotherapy for Burkitt lymphoma. *Journal of Pediatric Surgery*.

[13] Kaufman BH, Burgert EO, Banks PM (1987). Abdominal Burkitt’s lymphoma: role of early aggressive surgery. *Journal of Pediatric Surgery*.

[14] Gahukamble DB, Khamage AS (1995). Limitations of surgery in intraabdominal Burkitt’s lymphoma in children. *Journal of Pediatric Surgery*.

